# Supplementary material for: Mechanistic phylodynamic models do not provide conclusive evidence that non-avian dinosaurs were in decline before their final extinction
Source: Camb Prism Extinct. 2024 Apr 16;2:e6. doi: 10.1017/ext.2024.5 (PMC11895757; doi:10.1017/ext.2024.5)
Supplement: Allen et al. supplementary material [file S2755095824000056sup001.pdf]

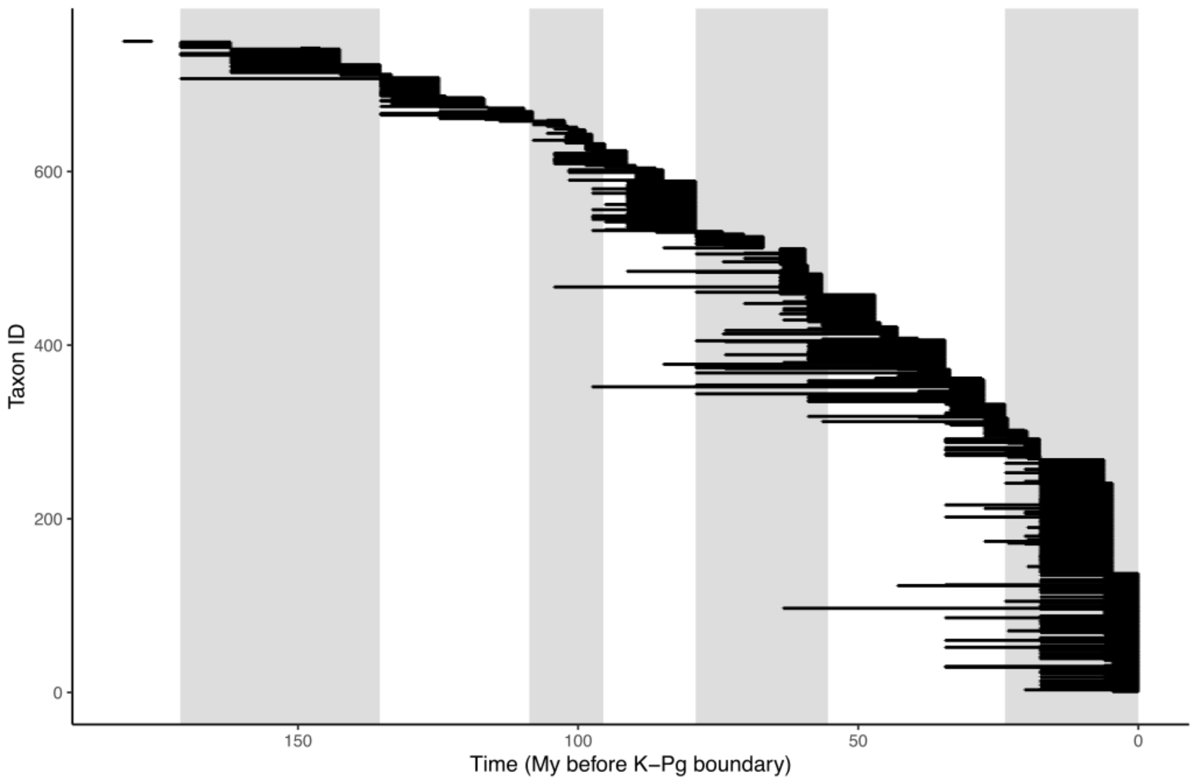

Supplementary Figure 1 The tip date priors used to timescale the phylogenies; each horizontal bar represents the age range of a species present in the largest (Lloyd2) phylogeny. The grey and white vertical bars correspond to the eight time bins used.

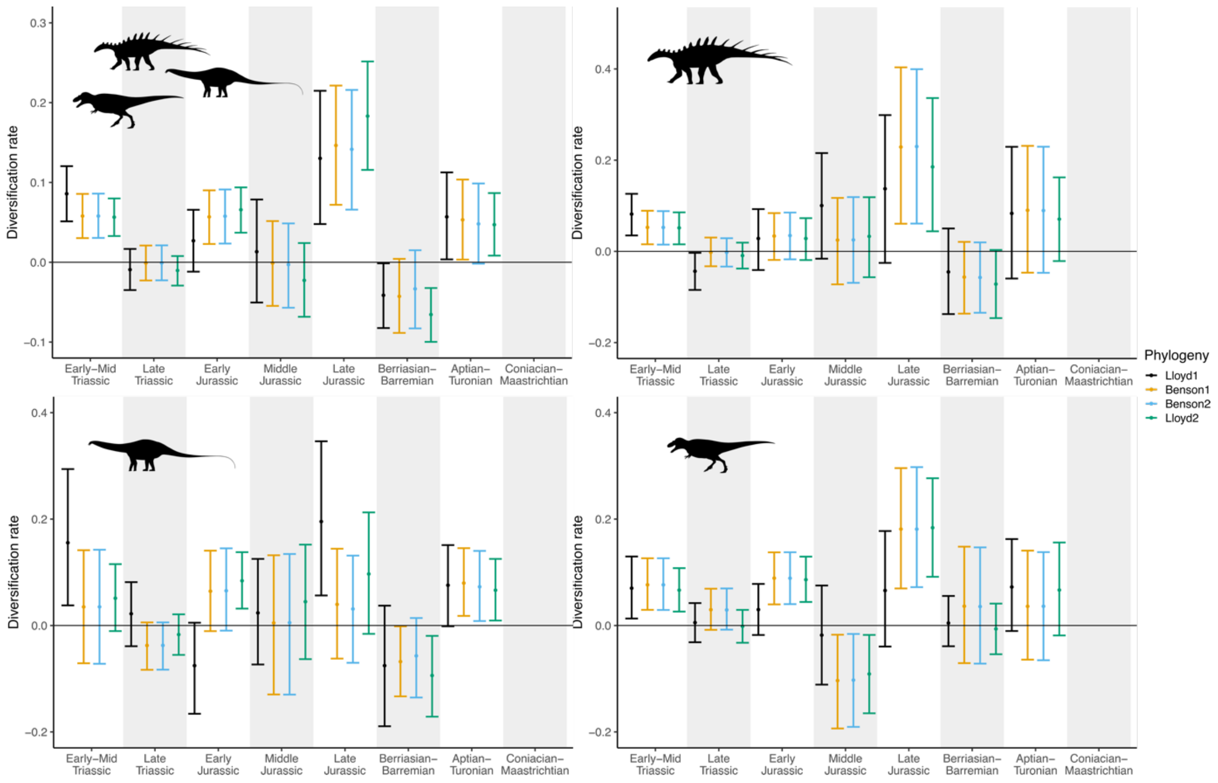

Supplementary Figure 2 Diversification rates estimated using the piecewise-constant fossilised birth-death skyline model. Matches Figure 2, except the y-axis is trimmed to exclude results from the Coniacian-Maastrichtian bin. Time moves forwards from left to right along the x-axis, with the K-Pg boundary at the end of the Coniacian-Maastrichtian bin. Estimates are shown for each of four phylogenies, ordered from smallest to largest. Points show the median values, and error bars indicate 95% highest posterior density. Dinosaur silhouettes for Ornithischia (top right), Sauropodomorpha (bottom left) and Theropoda (bottom right) are from Phylopic.

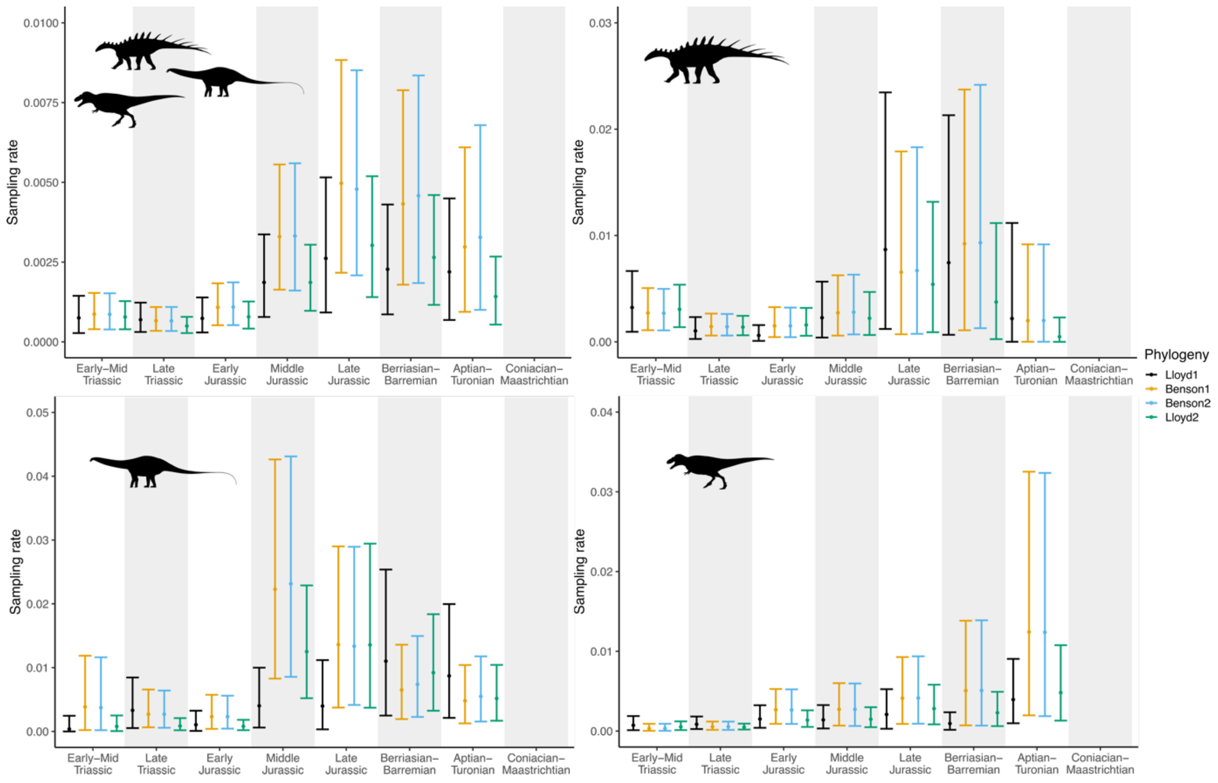

Supplementary Figure 3 Sampling rates estimated using the piecewise-constant fossilised birth-death model. Matches Figure 3, except the y-axis is trimmed to exclude results from the Coniacian-Maastrichtian bin. Time moves forwards from left to right along the x-axis, with the K-Pg boundary at the end of the Coniacian-Maastrichtian bin. Estimates are shown for each of four phylogenies, ordered from smallest to largest. Points show the median values, and error bars indicate 95% highest posterior density. Dinosaur silhouettes for Ornithischia (top right), Sauropodomorpha (bottom left) and Theropoda (bottom right) are from Phylopic.
